# Supplementary material for: The effect of hepatopancreas homogenate of the Red king crab on HA-based filler
Source: PeerJ. 2020 Feb 12;8:e8579. doi: 10.7717/peerj.8579 (PMC7023832; doi:10.7717/peerj.8579)
Supplement: Supplemental Information 5 [file peerj-08-8579-s005.pdf]

SUPPLEMENTARY

Table S1. Comparison of several HA-based fillers.

HA filler, manufacturer	Composition	The HA component is cross-linked with a cross-linking agent selected from the group consisting of
Juvederm Ultra 2, Allergan (USA)	24 mg/mL of HA, 0.3% lidocaine by weight of the composition, 90–95% high molecular weight (1–4 MDa) and low molecular weight (0.2–1 MDa) species. HA non-cross-linked from 89 to 96%, HA cross-linked from 4 to 11% [1, 2]	1,4-butanediol diglycidyl ether (BDDE), 1,4-bis(2,3-epoxypropoxy)butane, 1,4-bisglycidyloxybutane, 1,2-bis(2,3-epoxypropoxy)ethylene and 1-(2,3-epoxypropyl)-2,3-epoxycyclohexane, and 1,4-butanediol diglycidyl ether, or combinations thereof
Hyaluform filler deep, Laboratory THOSCANE (Russia)	25 mg/mL of HA, 2 MDa, 99.8% cross-linked HA and 0.2% non-cross-linked HA [3]	ethylene glycol diglycidyl ether, diethylene glycol diglycidyl ether, triethylene glycol diglycidyl ether, polyethylene glycol diglycidyl ether, propylene glycol diglycidyl ether, diglycidyl ether of 1,4-butanediol, and diglycidyl ether of 1,6-hexanediol
Revofil Ultra, Caregen (South Korea)	23 mg/mL of HA, Oligopeptide-72 (CG-Boostrin 1,000 ppm), Oligopeptide-50 (CG-Glamerin 300 ppm), 90% cross-linked HA, 10% non-cross-linked HA [4]	1,3-butilenglycol

Table S2. Comparison of several hyaluronidase products.

Hyaluronidase product, manufacturer	Composition	Origin
Lydaze, Microgen (Russia)	Hyaluronidase 1280 IU [5]	Cattle testicles
Longidaze, Petrovax Pharm (Russia)	Hyaluronidase 3000 IU. Hyaluronidase is conjugated with a high molecular weight synthetic support (40–100 kDa) with a ratio of enzyme to support of 1:1–5 [6]	Cattle testicles
Liporase, Caregen (South Korea)	Hyaluronidase 1500 IU	Ovine testicles

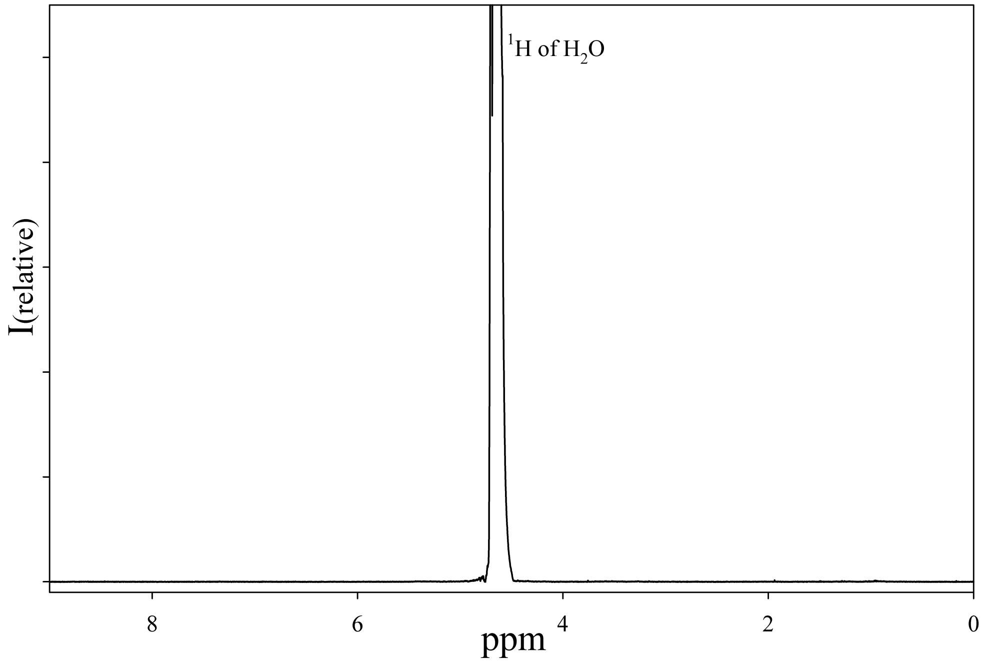

Figure S1. ^{1}H-NMR spectra of mix of purified HPC homogenate and phosphate buffer.

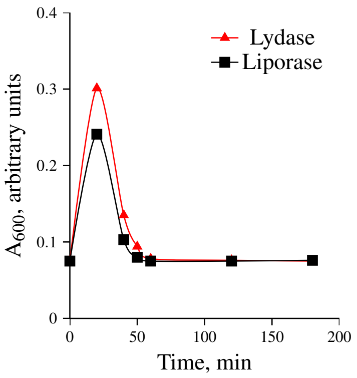

Figure S2. Study of Revofil Ultra filler HA using turbidimetric analysis during hydrolysis by Lydase and Liporase (the ratio of the total protein of the hyaluronidases (mg) to HA (mg) in the reaction mixture is 1:2).

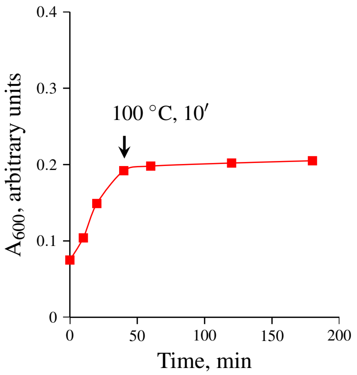

Figure S3. Study of Revofil Ultra filler HA using turbidimetric analysis during hydrolysis by HPC homogenate before and after increasing the temperature of the reaction mixture to 100 °C for 10 minutes.

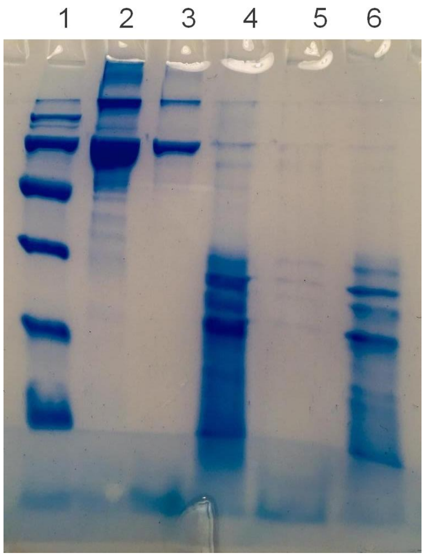

Figure S4. SDS-PAGE electrophoresis of HPC homogenate proteins before and after purification: 1, protein marker (from the bottom up: 14.4, 20.1, 30, 43, 67, and 94 kDa); 2 and 3, bovine serum albumin, 7 and 0.7 μg, respectively; 4, HPC homogenate before purification; 5, supernatant after salting out the homogenate with ammonium sulfate to 50% saturation during purification; 6, HPC homogenate after purification.

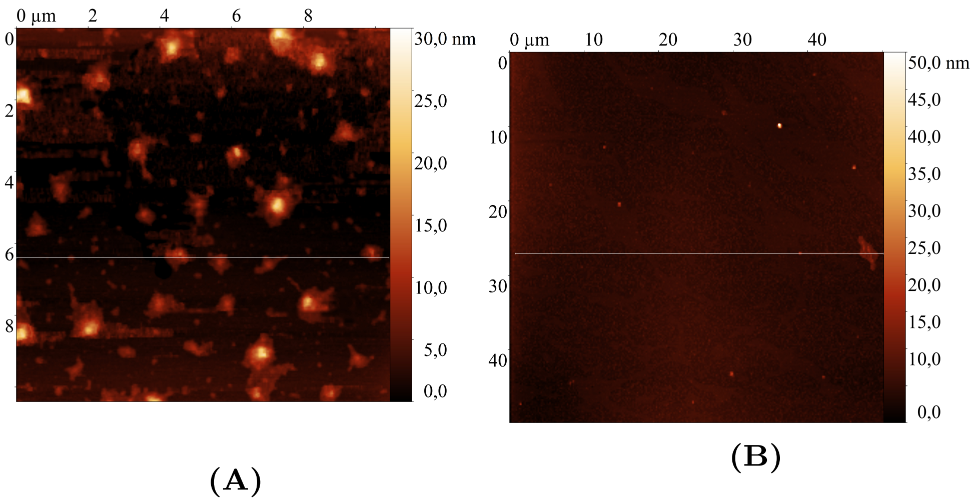

Figure S5. AFM images (obtained in tapping mode) of HPC homogenate diluted 21 times by phosphate buffer: **(A)** before purification and **(B)** after purification.

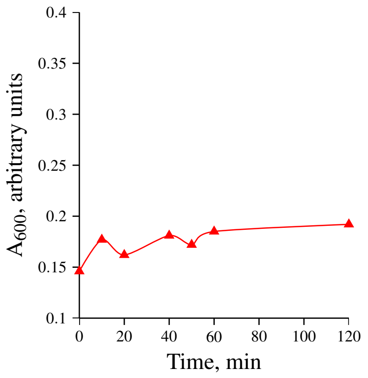

Figure S6. Hyaluform HA-based filler by turbidimetric analysis during incubation at 37 °C (0 min means that HA was not warmed before the beginning of the experiment).

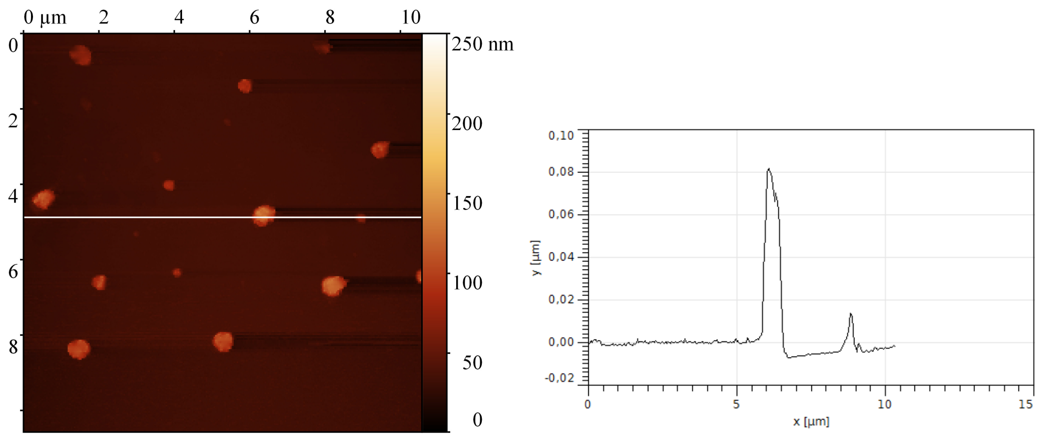

Figure S7. AFM images (obtained in tapping mode) of the Revofil Ultra HA-based filler.

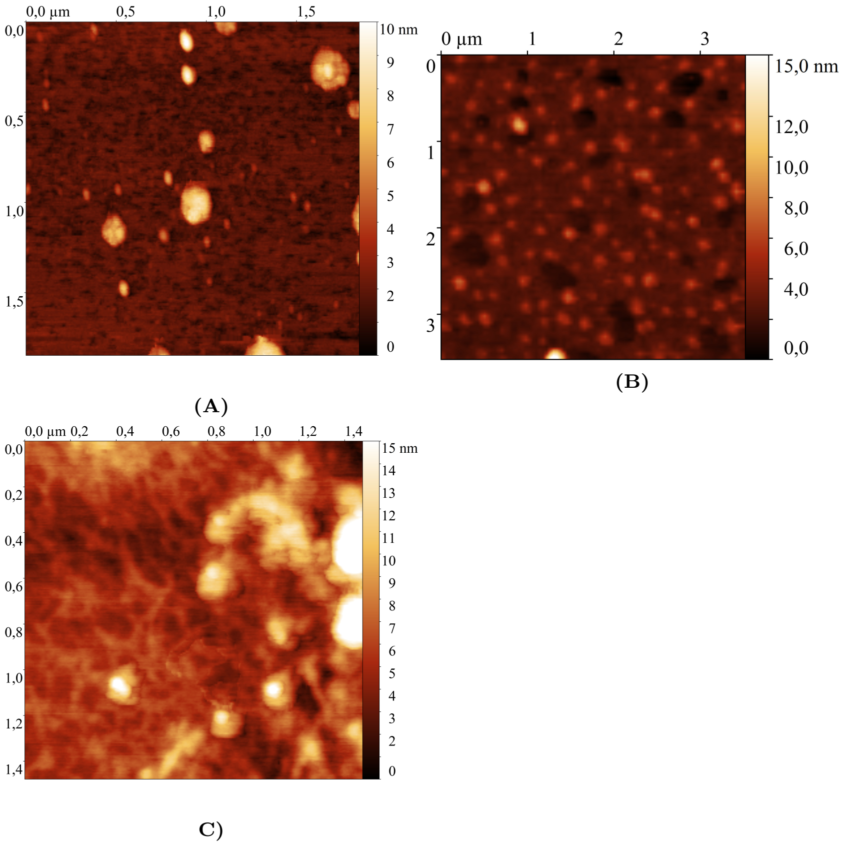

Figure S8. AFM images (obtained in tapping mode) of Hyaluform filler HA hydrolysates: **(A)** 5 min, **(B)** 40 min, and **(C)** 120 min of hydrolysis of Hyaluform filler HA using the purified HPC homogenate.

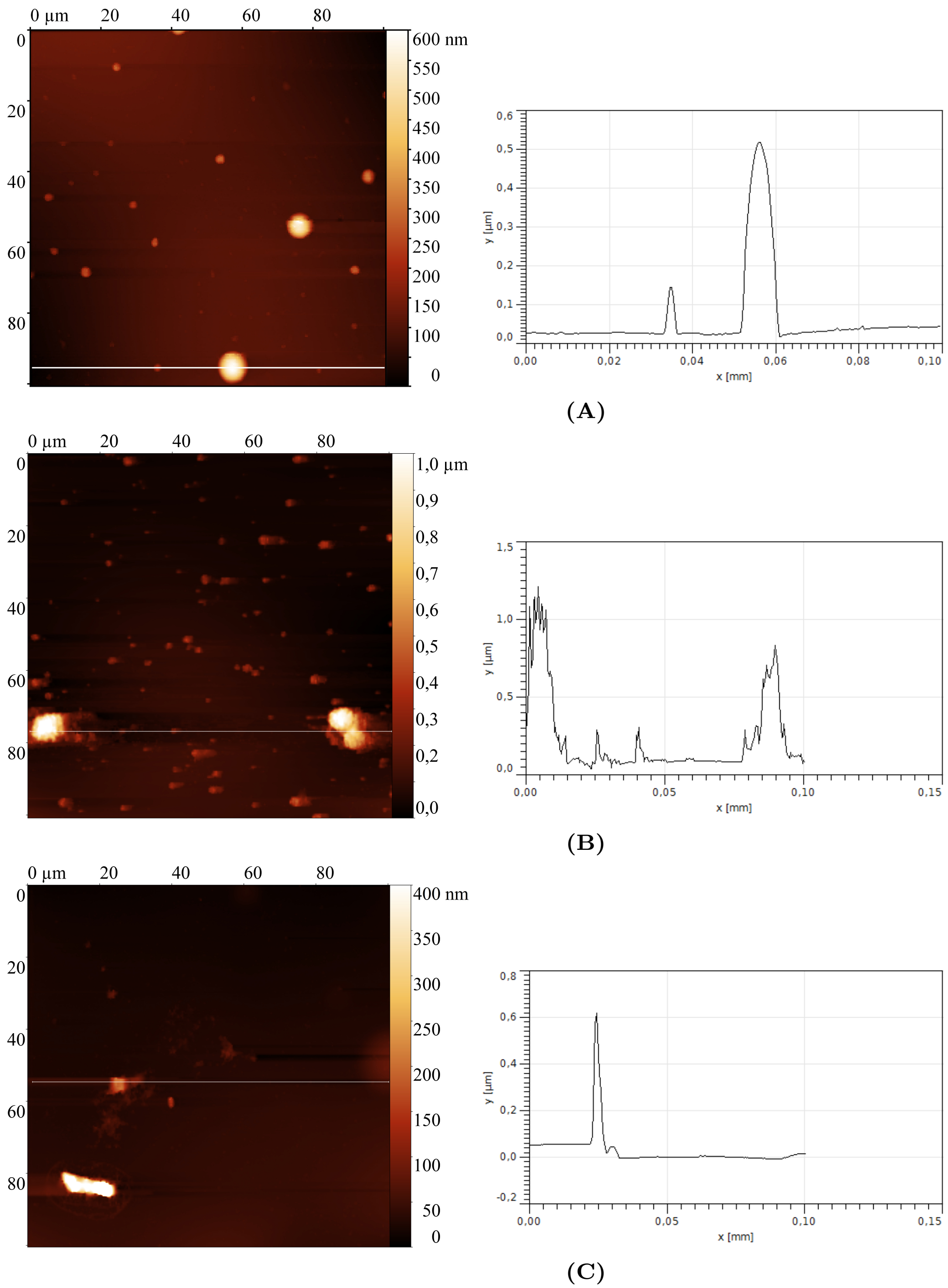

Figure S9. AFM images (obtained in tapping mode) of Hyaluform filler HA hydrolysates: (**A**) 5 min, (**B**) 40 min, and (**C**) 120 min of hydrolysis of Hyaluform filler HA using the purified HPC homogenate.

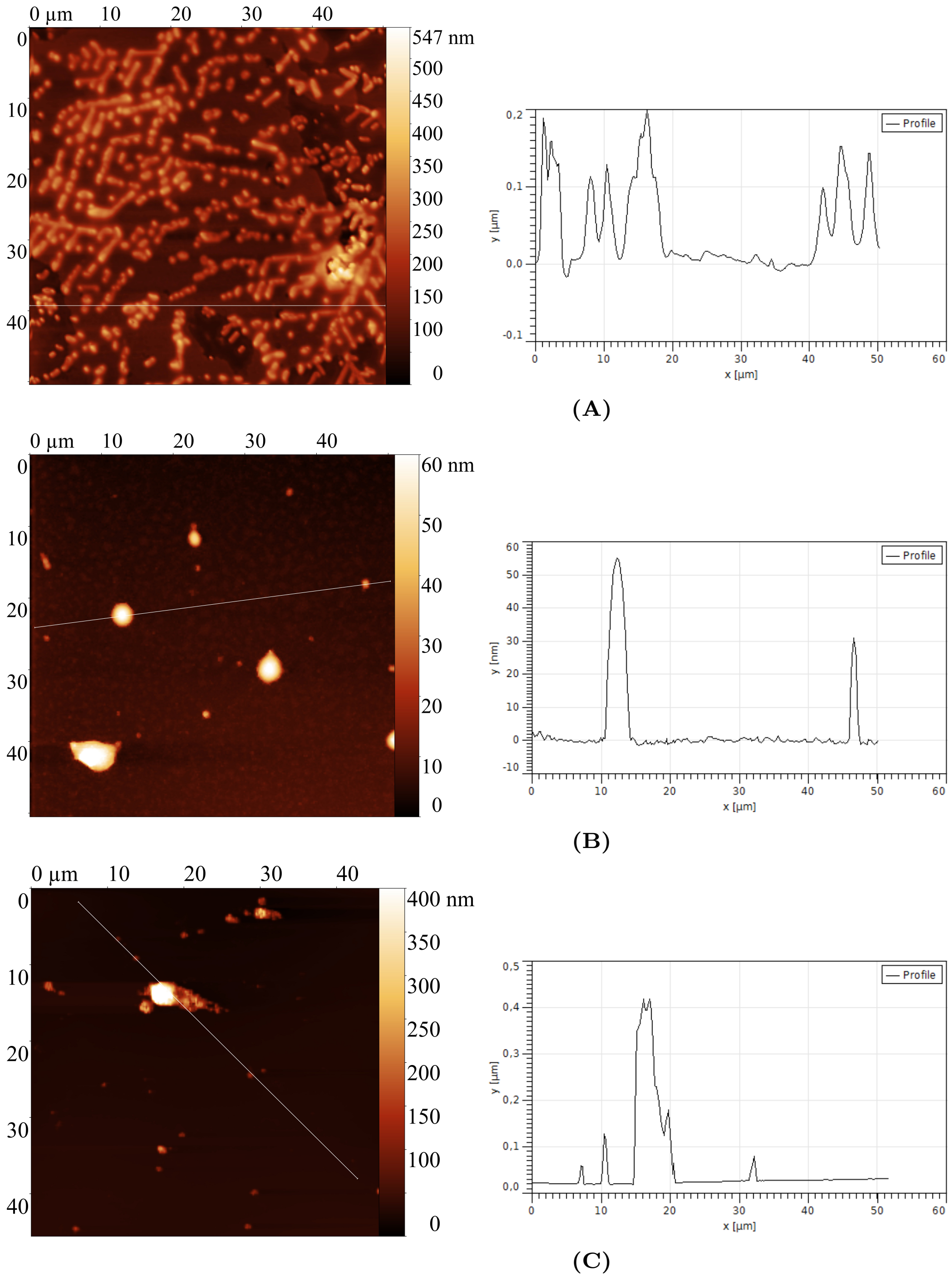

Figure S10. AFM images (obtained in tapping mode) of HA from rooster comb (**A**), 40 min (**B**) and 120 min (**C**) of hydrolysis of the HA using the purified HPC homogenate (50 × 50 μm field).

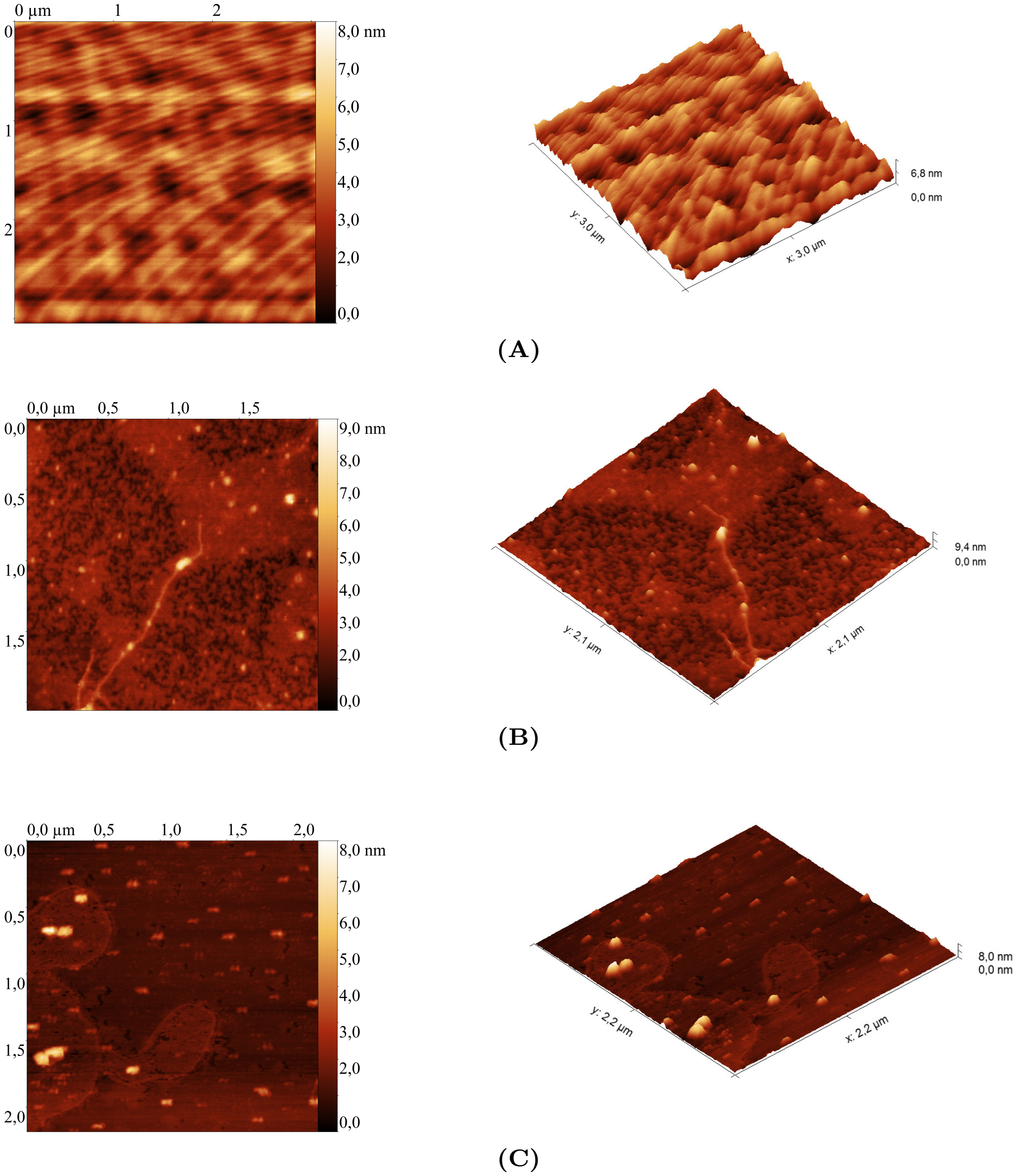

Figure S11. AFM images (obtained in tapping mode) of HA from rooster comb (A), 40 min (B) and 120 min (C) of hydrolysis of the HA using the purified HPC homogenate (3 × 3 μm or 2 × 2 μm field).

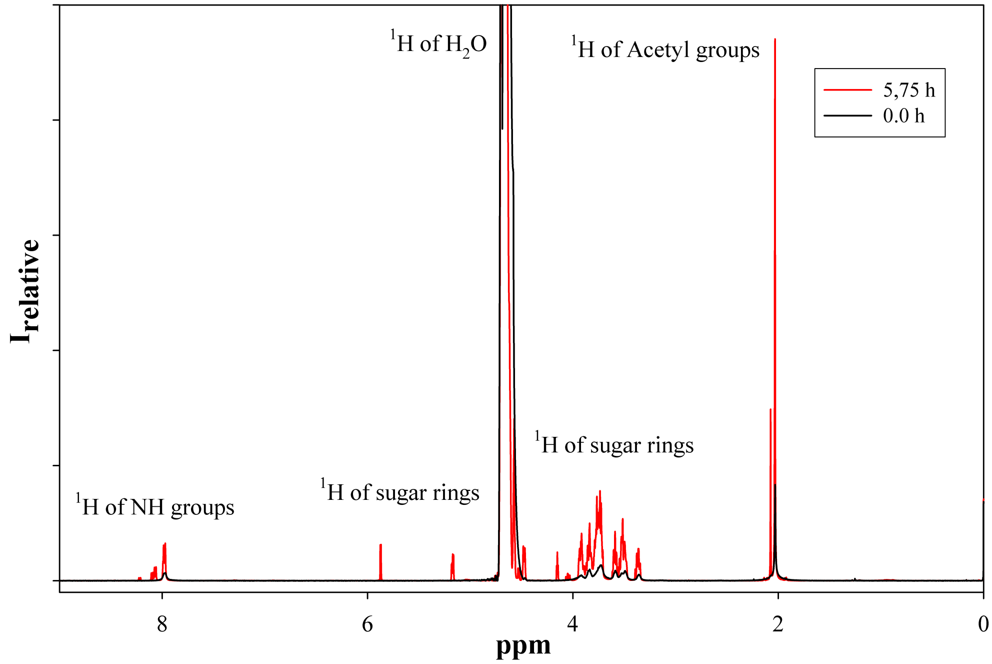

Figure S12. ^{1}H-NMR spectra of HA from rooster comb and its hydrolysis products after treatment by purified HPC homogenate. The black curve represents the HA from rooster comb, and the red curve represents the hydrolysis products after 5.75 h of incubation.

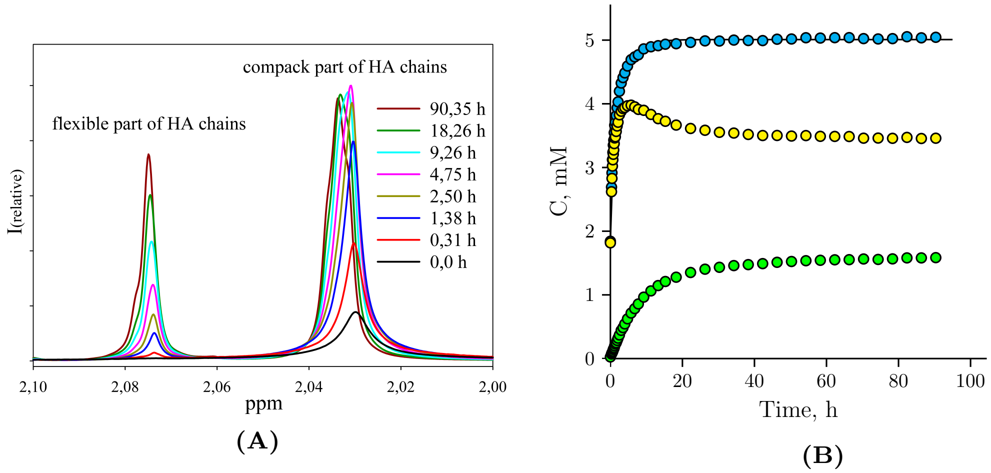

Figure S13. Hydrolysis of HA from rooster comb by purified HPC homogenate: **(A)** changes in the ^{1}H signal of the N-acetyl-D-glucosamine acetyl group of HA chains in ^{1}H-NMR spectra; **(B)** accumulation kinetics of the ^{1}H signal of the N-acetyl-D-glucosamine acetyl group of HA chains (integral under the peaks). The green marks represent the signal from the flexible part of the HA chains; the yellow marks represent the signal from the compact part of the HA chains; and the blue marks represent the total signals from both the flexible and compact parts of the HA chains.

REFERENCES

[1] Pierre F. Lebreton. Hyaluronic acid-based gels including lidocaine, February 2010.

[2] Pierre F. Lebreton. Hyaluronic acid-based gels including lidocaine. https://web.archive.org/web/20190821101512/https://www.lens.org/lens/patent/121-320-154-347-710/fulltext, 2009. Accessed: 2019-08-23.

[3] Vladimir Petrovich Volkov, Alexander Nikolaevich Zelenetskiy, Tatyana Anatolyevna Akopova, Vladimir Nikolaevich Khabarov, Mikhail Anatolyevich Selyanin, and Olga Nikolaevna Selyanina. Method for preparation of cured hyaluronic acid salts in water medium, January 2007.

[4] Simseongbo, Baksohyeon, Hongujin, and Choeeunho. Structure in which active material is inserted into de-differentiated plant protoplast, method for preparing same, and cosmetic composition containing same, July 2015.

[5] Yu. S. Nizhechik, I. A. Pestova, L. L. Ananicheva, and T. N. Tarasova. Method of high-quality preparation ”lidaza” preparing, June 2000.

[6] Arkady Vasilyevich Nekrasov, Temuri Musaevich Karapututze, Sergey Alekseevich Medvedev, Alexander Vladimirovich Kozyukov, and Nino Temurievna Karapututze. Method for preparing conjugate of hyaluronidase with derivatives of polyethylene piperazine and application of produced conjugate, April 2017.
